# Nano-Drug Delivery Systems for Bone Metastases: Targeting the Tumor–Bone Microenvironment

**DOI:** 10.3390/pharmaceutics17050603

**Published:** 2025-05-02

**Authors:** Mohamad Bakir, Ahmad Dawalibi, Mohammad Alaa Mufti, Ayman Behiery, Khalid S. Mohammad

**Affiliations:** 1 Department of Medicine, College of Medicine, Alfaisal University, Riyadh 11533, Saudi Arabia; mbakir@alfaisal.edu (M.B.); mmufti@alfaisal.edu (M.A.M.); 2 Department of Anatomy, College of Medicine, Alfaisal University, Riyadh 11533, Saudi Arabia; adawalibi@alfaisal.edu (A.D.); abehiery@alfaisal.edu (A.B.)

**Keywords:** bone metastases, nanoparticles in oncology, targeted drug delivery, bone-targeting nanoparticles, nanocarrier systems, nano-therapeutics for bone metastasis

## Abstract

Bone metastases are a prevalent and debilitating consequence of various cancers, including breast and prostate carcinomas, which significantly compromise patient quality of life due to pain, fractures, and other skeletal-related events (SREs). This review examines the pathophysiology of bone metastases, emphasizing the role of the bone microenvironment in tumor progression through mechanisms such as osteotropism and the dysregulated bone remodeling cycle. The primary focus is on the emerging nano-drug delivery systems (DDS) designed to target the bone microenvironment and improve the therapeutic index of anticancer agents. Current treatments, mainly comprising bisphosphonates and radiotherapy, provide palliative benefits but often have limited efficacy and significant side effects. Innovative strategies, such as bisphosphonate-conjugated nanoparticles and targeted therapies that utilize the unique bone marrow niche, are explored for their potential to enhance drug accumulation at metastatic sites while minimizing systemic toxicity. These approaches include the use of liposomes, polymeric nanoparticles, and inorganic nanoparticles, which can be functionalized to exploit the biological barriers within the bone microenvironment. This review also discusses the challenges and future directions for nano-DDS in clinical settings, emphasizing the need for multidisciplinary research to effectively integrate these technologies into standard care protocols.

## 1. Introduction

### 1.1. Epidemiology and Prevalence of Bone Metastases

Bone metastases in patients significantly increase the risk of death and reduce survival compared to that of patients before metastases, especially in those with prostate cancer. It is evident that the prevalence of bone metastases is highly contingent upon the type of primary cancer and the methodology used for detection. The high frequency of bone involvement in cancers such as prostate, lung, and breast cancers underscores the need for effective diagnostic and therapeutic strategies tailored to mitigate skeletal morbidity and improve patient outcomes [1,2].

### 1.2. Pathophysiology and Mechanisms of Bone Colonization by Metastatic Cells

The development of bone metastases is a multi-phase, complex process. In order to cause extravasation into the bone marrow space, tumor cells must separate from the primary tumor, enter the bloodstream (intravasation), evade immune detection, and stick to bone marrow capillaries [3,4]. Before reactivating inside the bone microenvironment, bone tumor cells first form micro-metastases, which may either develop into noticeable metastatic lesions or lie dormant for long periods. 

Invading tumor cells prepare the stroma in the pre-metastatic niche by generating chemicals that trigger responses in the bone microenvironment, which promotes tumor colonization and growth. Additionally, hematopoietic stem cell homing is impacted by bone resorption [5]. Tumor-cell-derived factors include osteopontin (OPN), which promotes bone marrow cell migration and tumor cell proliferation; heparanase (HPSE), which alters the extracellular matrix by reducing the length of the heparan sulfate chain, increasing bone resorption; and parathyroid-hormone-related protein (PTHrP), which promotes bone resorption and may also increase bone marrow chemokines like C-C motif ligand 2 (CCL2). 

Osteotropism is a phenomenon wherein tumor cells infiltrating the bone also produce signals that encourage ongoing recruitment to the bone microenvironment [6]. To promote tumor cell colonization in bone, αvβ3 integrin works in conjunction with matrix metalloproteinase-2 (MMP-2) and bone sialoprotein (BSP). Both tumor cell colonization and osteoclast-mediated bone resorption are facilitated by the receptor activator of nuclear factor kappa B (RANK) [7]. Osteoblasts and bone marrow stromal cells produce large amounts of CXC ligand 12 (CXCL12), also known as stromal-cell-derived factor 1 (SDF-1), which is a potent chemoattractant for hematopoietic stem cells (HSCs). The migration of malignant cells to bone is significantly aided by the expression of CXC receptor 4 (CXCR4) in cancer cells. Additionally, the bone microenvironment’s interactions between CXCL12 and CXCR4 lead to the upregulation of αvβ3 integrin, which improves cell adhesion.

### 1.3. Mechanisms of Tumor Cell Homing to Bone

Homing to bone is directed by chemokines, cytokines, and adhesion molecules, particularly the CXCL12/CXCR4 axis. CXCL12, secreted by osteoblasts and stromal cells, attracts CXCR4-expressing tumor cells. CCL2 further recruits monocytes/macrophages, promoting tumor survival. Integrins (αvβ3, α4β1), selectins, and CD44–hyaluronic-acid interactions mediate adhesion and extravasation into the bone marrow [8,9].

**Figure 1:**
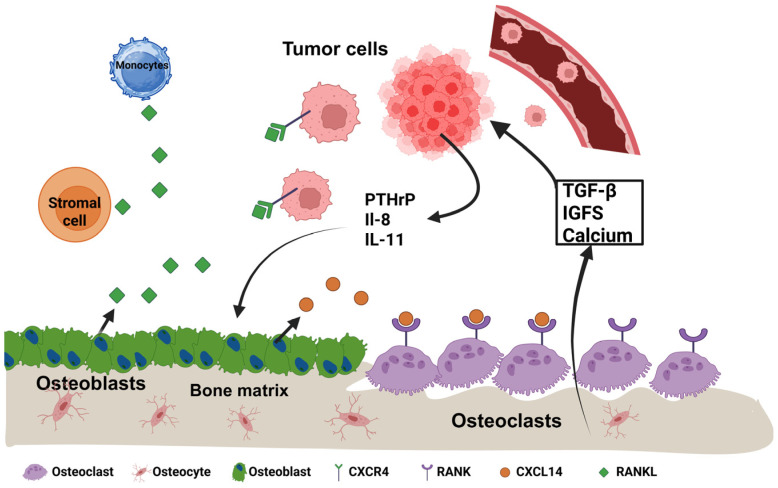
Bidirectional crosstalk that amplifies osteolytic bone metastasis. CXCL14, released by osteoblasts and stromal cells (orange), chemo-attracts circulating CXCR4^+^ tumor cells to bone. Lodged cancer cells secrete PTHrP, IL-8, and IL-11, prompting nearby osteoblasts (green) to produce RANKL (green diamonds). RANKL activates RANK on osteoclasts (purple), accelerating bone resorption. The resulting release of TGF-β, IGFs, and calcium from the matrix fuels tumor growth and survival, sustaining the “vicious cycle” of destruction. Osteocytes (pink) are shown in situ for anatomical context.

Upon invading the bone, tumor cells may enter a state of dormancy or proliferate, depending on the microenvironmental cues. Factors released during bone remodeling—such as TGF-β, IGFs, and BMPs—can reactivate dormant cells and drive tumor growth. Metastatic progression disrupts the osteoblast–osteoclast balance, forming a vicious cycle that sustains tumor expansion. In osteolytic metastases, tumor-derived PTHrP induces RANKL expression on osteoblasts, promoting osteoclast maturation and bone resorption. This releases TGF-β, IGFs, and calcium, further stimulating tumor growth (Figure 1). Pro-inflammatory cytokines (IL-6, IL-8, TNF-α) also enhance osteoclastogenesis and bone degradation. These dynamic interactions create a permissive metastatic niche, driving bone destruction, pain, and systemic disease spread [8,9].

## 2. Current Treatment Challenges

### 2.1. Conventional Treatments and Their Limitations

Bone malignancies present a medical problem because of their complexity, heterogeneity, malignant character, and the minimal advancements of the therapeutic approaches throughout the decades.

Despite its significant focus from researchers and medical professionals, therapy alternatives for bone cancers have stagnated since the late 1970s. Treatment selection is based upon multiple aspects, including the cancer type and features, if the disease is localized or widespread, the presence of extraskeletal metastases, past treatment history, signs and symptoms, and overall health status [10].

Despite the variety of treatments available for bone metastases, selecting the most effective one can be challenging. Both minimally invasive treatment and open surgery have limits and may not be appropriate for many patients. Surgical techniques are intended to repair broken bones and alleviate pain.

Nevertheless, any surgery involves potential hazards, and not all patients qualify as acceptable candidates due to criteria such as overall health status and the degree of metastatic dissemination, as well as other side effects and complications associated with any surgical procedure [11].

Also, the surgical procedure options and benefits are limited for patients suffering from large or multiple bone metastasis locations [9,12].

Systemic chemotherapy attacks growing cancer cells, although its effectiveness against bone metastases is frequently restricted. Challenges encompass multidrug resistance, tumor recurrence, and significant side effects, which may impair patient quality of life [10]. Significant chemotherapy-associated adverse effects identified in multiple studies encompass nausea, vomiting, fatigue, anorexia, gastrointestinal disturbances, hair loss, pain, and anemia, all of which profoundly affect patients’ quality of life [13].

Radiotherapy has become the preferred treatment for bone metastases and is widely utilized in clinical settings. Nonetheless, it is less efficient in people with pathological fractures and spinal instability. Research indicates that radiation is unsuccessful in around one-third of individuals with bone metastases [14]. Meta-analysis studies indicate that over 40% of patients experienced around a 50% drop in pain levels about one month post-radiotherapy, whereas fewer than 30% achieved total pain relief [15]. Radiotherapy typically requires four to six weeks to achieve its maximum efficacy; however, a considerable number of patients with bone metastases possess a limited survival prognosis [16]. There are many types of cell death induced by radiation. However, radiation therapy primarily induces cell death by apoptosis or mitotic catastrophe [17]. The advantages of stereotactic body radiation therapy (SBRT) over conventional palliative radiotherapy in treating bone metastasis discomfort are highlighted in this review.

Due to the methodological limitations of the available evidence, substantial randomized studies are now critically needed to assess this advantage more accurately [18]. The side effects of radiotherapy are many. However, the treatment areas and potential adverse effects are based on the specific body region undergoing radiation therapy [19].

Up to 75% of cases of metastatic breast cancer include bone metastases, which frequently result in skeletal-related events (SREs). Osteoclast inhibitors, particularly denosumab, are first-line therapies that are more effective than bisphosphonates, such as zoledronate, in preventing SREs. However, both carry risks, including osteonecrosis of the jaw and hypocalcemia. Bisphosphonates also pose a risk of nephrotoxicity, necessitating serum creatinine monitoring before each dose; moreover, their use is contraindicated in patients with a creatinine clearance of less than 30 mL/min. Additionally, bisphosphonates can cause acute-phase reactions and, unlike denosumab, lack direct antitumor effects [20].

### 2.2. The Need for Targeted, Efficient, and Less Toxic Therapeutic Strategies

To lessen side effects and systemic toxicity and address resistance mechanisms, therapies that selectively target metastatic cells within the bone microenvironment are necessary, as shown by the deficiencies of current treatments. Nanotechnology offers effective solutions by developing targeted drug delivery systems (DDS), increasing the efficacy of therapy by enhancing drug concentration at the tumor location, and minimizing side effects. Nanocarriers can be designed to target medications specifically to bone metastases, enhancing drug concentration at the tumor location while minimizing systemic exposure. Numerous nanomaterials, such as liposomes, polymers, and inorganic nanoparticles, have been investigated for this application [21,22].

Nanomaterial-based drug delivery systems (DDSs) are gaining increasing acceptance as a viable therapeutic option for various tumor types, owing to their ability to overcome biological barriers and enhance drug delivery efficiency. Recent studies on nanoparticle (NP)-based drug delivery systems have demonstrated enhancements in the permeability and retention (EPR) effect in bone metastasis. Drug delivery systems for bone metastasis therapy frequently focus on hydroxyapatite and αvβ3 integrin, which are distinctive components found in bone [12]. Using NP to treat metastases will prolong circulation and prevent the active medication from being cleared or degraded too soon, consequently enhancing drug buildup at the metastatic site [23]. Bisphosphonates (BPs) exhibit a strong affinity for hydroxyapatite, enabling their attachment to NPs [8,12]. In addition to targeting tumors and having long half-lives in the body, they also enhance the immunosuppressed tumor microenvironment and activate tumor-killing T-cells. This dual role improves therapeutic efficacy against tumors while minimizing the required dosage of medications, as well as decreasing the toxic adverse effects on normal organs [12,24,25].

Modifying peptides targeting the NP surface enables site-specific drug release, ensuring that toxicity is confined to tumor tissue while sparing healthy tissues. Through MMP2/9-triggered cleavage, researchers created a multifunctional and multiresponsive superparamagnetic iron oxide nanoparticle system that selectively targets bone metastases sites to release furin-inhibitory peptides, thereby exerting anticancer and anti-osteoclastic effects [12,26]. Chemotherapeutic drugs and osteoclast inhibitors combined in a single nanocarrier may efficiently target bone resorption and tumor growth, thereby enhancing treatment outcomes [27].

### 2.3. Rationale for Nano-Based Drug Delivery

Because they are hydrophobic, many anticancer drugs used to treat bone metastases have poor water solubility and low bioavailability. Nanocarriers can encapsulate hydrophobic medications to increase their stability and solubility in biological fluids [12]. Conventional chemotherapy frequently results in the extensive dissemination of medications, impacting both malignant and healthy tissues, thereby inducing considerable adverse effects. Nanocarriers can address this problem by delivering drugs more selectively to target sites, thus reducing the exposure of healthy tissues and minimizing systemic toxicity. Nanoparticle-mediated delivery of doxorubicin has been demonstrated to reduce cardiotoxicity and enhance drug pharmacokinetics, leading to improved therapeutic outcomes in bone metastases [28].

Nanocarriers can be designed to identify and attach to particular cells or tissues, facilitating targeted drug delivery. In bone metastases, nanocarriers can be engineered with bone-targeting agents like bisphosphonates or alendronate, which exhibit a strong affinity for the bone mineral hydroxyapatite. This target increases drug accumulation at bone metastatic locations, enhancing therapeutic efficacy. Alendronate-conjugated nanoparticles have demonstrated efficacy in delivering paclitaxel to bone metastases, inhibiting tumor growth and mitigating bone loss [28]. Furthermore, nanocarriers can be engineered to target overexpressed cancer cell receptors, including integrin αVβ3 and CD44. Functionalizing nanoparticles with ligands or antibodies that target these receptors enables selective binding and internalization by tumor cells, thereby enhancing the precision of drug delivery. Nanoparticles designed to target integrin β3 have been created to deliver doxorubicin specifically to bone metastases of mammary carcinoma, thereby improving chemotherapy delivery to breast cancer cells [12].

The utilization of nanocarriers for drug delivery has advanced considerably in the last decade, as it promises to overcome the constraints of traditional formulations. Pharmaceutical nanocarriers provide a viable substitute for traditional transdermal drug delivery systems by addressing the limitations inherent to this delivery method. Nanocarriers offer an effective and tailored approach to biomolecular interactions, mitigating treatment-related adverse effects. Nanoparticles, when employed as drug delivery systems, offer numerous advantages, including enhancing the solubility of poorly water-soluble medications, modifying pharmacokinetics, prolonging the drug half-life by reducing immunogenicity, increasing bioavailability, and slowing down drug metabolism. However, the stratum corneum is a crucial barrier to drug permeation in transdermal delivery, but the application of nanocarriers for transdermal drug delivery has notably advanced to surmount this obstacle [29].

## 3. Biological and Physiological Considerations for Targeting Bone Metastases

### 3.1. The Bone Microenvironment

The bone structure is highly vascularized, and the valveless Batson’s plexus, characterized by its sluggish blood flow and substantial vessel size, serves as an optimal location for the colonization of invasive neoplastic cells. Upon colonization of the bone by tumor cells, stromal cells, osteoclasts, and transient cells within the bone microenvironment can promote tumor development and spread via several pathways and mechanisms [30,31]. Conventional drug delivery technologies have specific constraints, including drug instability, limited solubility, and extrinsic metabolism. Conversely, drug-loaded nanoparticles provide benefits including controllability, substantial loading capacity, and protective characteristics, which can address these challenges [30,32]. The aberrant structure of tumor-induced neovasculature might result in uneven blood flow and regions of hypoxia, complicating consistent medication administration [30].

Drug delivery systems using nanoparticles can be divided into passive and active targeting systems depending on their targeting methods. The passive accumulation of nanoparticles in tumor tissues, mostly due to the EPR effect, is the foundation of passive targeting. This phenomenon arises from the leaky vasculature and inadequate lymphatic outflow in tumor tissues, facilitating the accumulation of nanoparticles at the tumor location [30,33]. The size of nanocarriers is a crucial factor influencing their penetration and retention in tumors, as it is constrained by the interstices in tumor blood vessels, which generally range from 200 to 800 nm. The excretion pathways of nanomedicines are greatly influenced by their dimensions, as follows: particles bigger than 500 nm are typically eliminated by the reticuloendothelial system, whereas particles less than 6 nm are frequently eliminated by renal filtration. Consequently, nanocarriers work best in the 20 to 200 nm size range. Conversely, active targeting involves attaching targeting molecules to the surfaces of nanocarriers, allowing them to recognize specific cells selectively. Ligand-modified nanocarriers enhance receptor-mediated endocytosis by attaching to cell surface receptors [30]. Blood vessels formed by tumors are often irregular, exhibiting inconsistent sizes and disorganized morphology. This abnormal vasculature may result in an irregular blood flow and increased interstitial fluid pressure (IFP), obstructing the efficient delivery and infiltration of nanoparticles into the tumor site [33]. Increased IFP in tumors arises from permeable blood vessels and a compact extracellular matrix, which impede nanoparticle infiltration and lead to inadequate therapeutic applications of nanoparticles due to the variability of the EPR effect [34].

Hyperthermia (HT) serves as an effective method to enhance the EPR effect by increasing tumor blood flow (TBF) and vascular permeability and reducing IFP and altering the architecture of the ECM. Moreover, the HT-triggered intravascular release method can surmount the EPR effect. Unlike pharmaceutical methods, HT is safe and can target cancerous regions. Additionally, the direct anticancer capabilities of HT increase the efficacy of anticancer medications contained in nanoparticles [34].

### 3.2. Tumor Biology in Bone

Malignant bone tumors with osteolytic lesions represents a clinical challenge. Bone-targeting nanomedicines, such as those utilizing alendronate and pamidronate, lack selectivity between healthy bone and osteolytic lesions due to the high affinity of bisphosphonates for all bone tissues. A promising alternative is a carboxyl-terminated dendrimer, which preferentially delivers therapeutic nanoparticles to osteolytic lesions. Its high carboxyl group density provides strong bone-binding properties, allowing platinum nanoparticles to accumulate primarily at osteolytic sites rather than healthy bone. Photothermal therapy using this dendrimer effectively inhibits bone tumors and osteolysis with minimal cytotoxicity and hematologic toxicity. This approach offers a targeted and efficient strategy for treating malignant bone tumors [35].

A study indicated that silica-based nanoparticles exhibit inherent biological activity concerning the skeleton, facilitating the differentiation of bone-forming osteoblasts while suppressing the differentiation of bone-resorbing osteoclasts. The enthusiasm for nanomedicine stems partly from its virtually limitless potential to modify physicochemical properties, such as size, composition, and surface charge. Spherical silica nanoparticles of varying sizes (50–450 nm), diverse surface properties (OH, CO_2_H, NR_4_^+^, and mNH_2_), and distinct compositions (silica, gold, and polystyrene) were synthesized and assessed for biological activity against skeletal cells. Compositional and dimensional variables predominantly influenced osteoblast activity, while osteoclasts were primarily affected by variations in surface properties. This research demonstrates the nanoparticle-mediated inhibition of Nfatc1, a crucial transcriptional regulator of osteoclast differentiation, thereby revealing a novel mechanism of action. This study emphasizes that, in the design of bioactive nanoparticles, it is essential to consider both the numerous manipulable physical properties and the characteristics of the target cells, as both significantly influence the biological outcome [36].

Malignant bone tumors with aggressive osteolysis remain challenging to treat. Nanomedicine offers a promising solution, as demonstrated by a multifunctional melanin-like nanoparticle designed for targeted chemo-photothermal therapy. This system, based on alendronate-conjugated polydopamine (PDA-ALN), exhibits strong affinity for hydroxyapatite and enhanced photothermal properties. Adding Fe ions (PDA/Fe-ALN) improved MRI contrast and preferentially accumulated at osteolytic lesions compared to non-targeted PDA. The encapsulation of the chemotherapeutic SN38 enabled controlled release under near-infrared light and acidic conditions. This dual-action platform effectively suppressed tumor growth and reduced bone degradation at a moderate temperature (~43 °C), highlighting its therapeutic potential in treating malignant bone tumors [37].

Another study discusses phytic acid (PA), a naturally occurring substance with anticancer and bone-targeting qualities. While maintaining the inherent anticancer qualities of PA and the photothermal effects of platinum nanoparticles, the PA-capped platinum nanoparticles demonstrated a remarkable affinity for hydroxyapatite both in vitro and in vivo. When exposed to near-infrared light, the PA-capped nanoparticles efficiently inhibited the growth of bone tumors and tumor-associated osteolysis, and they showed a four-fold higher accumulation in osteolytic lesions than sodium-citrate-templated nanoparticles [38].

## 4. Overview of Nano-Based Drug Delivery Platforms

**Figure 2:**
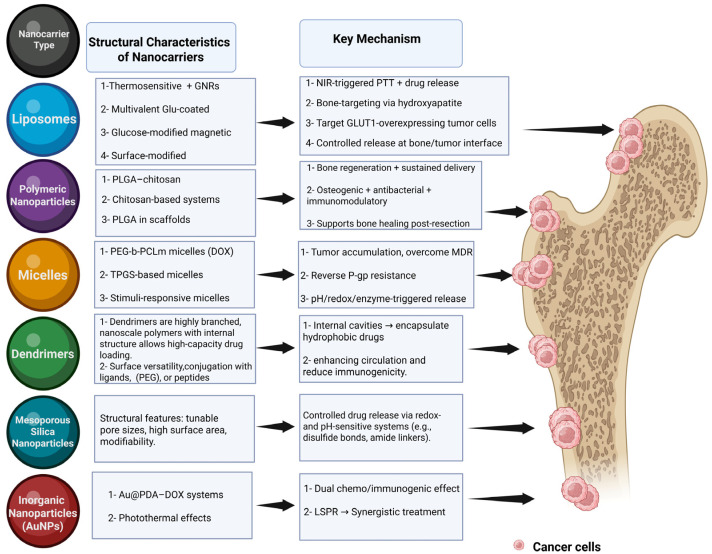
Engineered nanocarrier platforms and their therapeutic logic for treating bone-located malignancies. Six major nanocarrier classes are depicted with their design features and therapeutic mechanisms at the bone–tumor interface. Liposomes functionalized with gold nanorods (GNRs) enable NIR photothermal therapy (PTT)-triggered drug release, while glutamate or glucose modifications support bone- or tumor-targeting via hydroxyapatite or GLUT1 binding. Polymeric nanoparticles, such as PLGA- or chitosan-based systems, deliver osteogenic, antibacterial, and immunomodulatory cues via sustained release from bone scaffolds. Amphiphilic micelles (e.g., PEG-b-PCLm- and TPGS-based ones) concentrate doxorubicin (DOX), overcome P-gp-mediated multidrug resistance (MDR), and enable stimuli-responsive drug liberation. Dendrimers encapsulate hydrophobic drugs and prolong circulation through PEGylation or peptide conjugation. Mesoporous silica nanoparticles employ tunable pore structures and redox- or pH-responsive gatekeepers for on-demand release. Gold nanoparticles (Au@PDA) combine chemotherapy with immunogenic cell death and photothermal effects via localized surface plasmon resonance (LSPR). Arrows indicate how each system targets or is retained within bone metastases (pink), disrupting tumors and promoting bone repair. Figure created with BioRender.com. Abbreviations: PLGA, poly(lactic-co-glycolic acid); PCL, poly(ε-caprolactone); PEG, polyethylene glycol; TPGS, d-α-tocopheryl polyethylene glycol succinate; DOX, doxorubicin; LSPR, localized surface plasmon resonance.

The rapid evolution of nanotechnology has produced a diverse array of carrier architectures, ranging from classical liposomes and polymeric nanoparticles to sophisticated dendrimers, mesoporous silica, metal/metal oxide particles, and hybrid organic–inorganic constructs that collectively redefine how therapeutic agents can be delivered to diseased tissues. This section surveys these platforms, progressing from “conventional” to “advanced”, and highlights the physicochemical design principles (size, surface charge, porosity, and stimuli responsiveness) that influence circulation half-life, biodistribution, drug-loading capacity, and on-demand release (Figure 2). Emphasis is placed on strategies that enhance bone tropism, such as bisphosphonate or peptide ligands, magnetic guidance, and pH/redox-triggered gatekeepers, because overcoming the unique biological and mechanical barriers of the skeletal microenvironment is pivotal for the effective treatment of bone metastases. By comparing each nanocarrier class’s strengths, limitations, and translational status, this section lays a foundation for selecting engineering platforms that maximize the therapeutic index while minimizing systemic toxicity in oncologic applications (Table 1).

Comparative effectiveness of organic vs. metal nanocarriers. Organic systems (liposomes, polymeric NPs, micelles, and dendrimers) typically achieve higher drug-loading per mass of carrier (5–20 wt %) and superior biodegradability, making them ideal for sustained cytotoxic delivery; however, their passive bone accumulation rarely exceeds 5% ID g^−1^. By contrast, metal and metal-oxide nanoparticles (Au, Pt, and SPIONs) provide unique photothermal- or magnetic-guidance functions that raise local drug release and imaging precision and—as shown in several mouse models—double the tumor-to-bone drug ratio relative to organic analogues, albeit with concerns over long-term metal clearance.

The classes of cancer-active nanoparticles and their therapeutic trade-offs are as follows:

- Liposomal and polymeric carriers: clinically validated (e.g., Doxil^®^, Abraxane^®^‡), high drug-loading and biodegradability, but prone to leakage and require cold-chain logistics;
- Polymeric micelles: excellent solubilisers for hydrophobics and low critical micelle concentration, yet can disassemble under shear forces in the bloodstream;
- Dendrimers: multivalent targeting and tunable size; hurdles are costly synthesis and cationic-surface cytotoxicity;
- Mesoporous silica (MSNs): record surface area supports gate-controlled release; long-term RES retention and PEG-dependent uptake variability remain concerns;
- Metal/metal-oxide NPs (Au, Pt, TiO_2_): add photothermal, photodynamic, or radiosensitizing functions and permit image-guided therapy; key disadvantages are metal-ion persistence and potential off-target heating;
- SPIONs: enable MRI-visible magnetic targeting and magnetothermal release; heterogeneous tumor penetration and sparse clinical safety data are limiting factors;
- Hybrid organic–inorganic systems: combine biodegradability with multimodal imaging/therapy; synthetic complexity and regulatory path are still evolving.

### 4.1. Conventional Nanocarriers

- Liposomes

**Figure 3:**
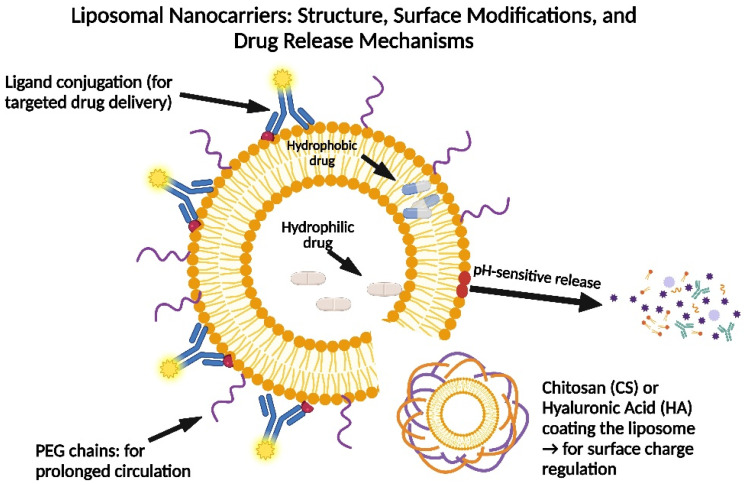
Structure and surface modifications of liposomal nanocarriers for bone metastasis therapy. This diagram illustrates the key components of liposomal nanocarriers, including hydrophilic and hydrophobic drugs, PEG chains for prolonged circulation, and surface modifications such as chitosan or hyaluronic acid for charge regulation. The figure also highlights ligand conjugation for targeted drug delivery and pH-sensitive release mechanisms.

The aqueous core of liposomes is surrounded by one or more phospholipid bilayer membranes, forming nanoscale spherical vesicles. Liposomes have been widely used in many nanomedicine and biomedical applications, such as drug delivery nanocarriers, nutraceuticals, immunoassays, tissue engineering, clinical diagnostics, and theranostic formulations, because of their remarkable biocompatibility, versatility in chemical composition, ease of synthesis, and varied structural properties (Figure 3). Liposomes play a particularly important role in drug administration because they increase regulated distribution and activity, both in vitro and in vivo, and reduce toxicity and adverse effects in encapsulated medications. Due to these applications, significant attempts were made to scale up the formation processes for suitable industrial development. Despite improvements in conventional techniques and the development of novel liposome preparation methods, their intrinsic vulnerability to mechanical and chemical influences leads to serious problems with low colloidal stability and reduced cargo molecule entrapment efficiency [39].

The biodistribution, medication interactions, and overall therapeutic efficacy of nanoparticles are all influenced by their zeta potential. A study demonstrated a simple and cost-effective method to modulate liposomal surface charge through proton competition between cationic chitosan (CS) and anionic hyaluronic acid (HA). While CS or HA coatings improve stability, the ratio of charged lipids (such as DOTAP or DOPS) is traditionally used to adjust the liposomal charge. These coatings also enable pH-responsive surface charge tuning without altering liposome size. The resulting nanocarriers showed minimal cytotoxicity in vitro and effectively delivered antitumor agents. Tailoring liposomal charge can thus support targeted therapy, high drug encapsulation, and controlled release, offering a flexible platform for drug delivery [40].

Liposome matrices face challenges like drug leakage and instability. To address this, surface modification via polymer coating or incorporation has been applied. Curcumin was encapsulated within the liposome core of three nanocapsules (N1, N2, and N3) made using DMPC, PDDA polymer, and silica nanoparticles through layer-by-layer assembly using the thin film hydration process. Fluorescence spectroscopy, TGA, DLS, XRD, SEM, and zeta potential analysis were among the characterization methods used. For N1, N2, and N3, the fluorescence intensity of curcumin increased by roughly 25-, 54-, and 62-fold, respectively. Better antitumor effectiveness, drug loading, and encapsulation were attained, with curcumin release decreasing as surface layering increased. This enhanced performance is attributed to electrostatic interactions that stabilize curcumin within the nanocapsules [41].

A study created a straightforward nanoplatform that combines gold nanorods (GNR) and thermosensitive liposomes (TSL) to treat bone metastases using improved chemotherapy and GNR-assisted photothermal therapy (PTT). TSLs were made to be stable in physiological settings and responsive to PTT-induced moderate hyperthermia. Simple incubation was used to mix the drug-loaded TSLs with GNR. Through PTT induction, thermosensitive drug release, and improved drug sensitivity, NIR irradiation of the nanoplatform successfully decreased tumor cell survival and migration. The nanoplatform effectively transported medications and GNR to metastatic locations in a mouse bone metastasis model, releasing them upon localized NIR irradiation. Through the removal of tumor cells and the restoration of the proper ratio of osteoclasts to osteoblasts, this method enhanced survival, reduced discomfort, and conserved bone structure. This liposome–GNR system is a promising treatment option for bone metastases due to its ease of use and efficacy [42]. In another study, the preparation of liposomes was investigated. These liposomes are a series of innovative multivalent glutamic hexapeptide derivatives that were synthesized and designed as ligands. They are capable of effectively conveying paclitaxel (PTX) to bone. They produced liposomes and assessed their hemolysis, zeta potential, stability, particle size, encapsulation efficiency, and release kinetics. The results showed significant bone-targeting efficacy for the coated liposomes, PTX-Glu61-Lip, PTX-Glu62-Lip, PTX-Glu63-Lip, and PTX-Glu65-Lip. As evaluated both in vitro and in vivo, PTX-Glu65-Lip showed greater targeting ability and anti-bone-metastasis efficacy when compared to other coated liposomes [43].

Using a novel glucose derivative that targets GLUT1-overexpressing cancer cells, a glucose-modified magnetic liposome (G-MLip) was created to improve the transport of paclitaxel (PTX) to bone metastases. PTX-loaded G-MLip (PTX-G-MLip) was made using ultrasonic and film hydration techniques. In contrast to free PTX and non-targeted liposomes, PTX-G-MLip demonstrated noticeably more drug accumulation in bone metastatic lesions when exposed to a magnetic field. These results demonstrate the potential of glucose-modified magnetic liposomes as a targeted medication delivery method for the treatment of bone metastases [44].

Recent developments have addressed issues with drug loading and release restrictions, and liposome-based drug delivery systems provide increased safety and efficacy. Co-solvents, surfactants, and optimized lipid compositions all increase loading capacity while preserving stability. Multifunctional, stimuli-responsive liposomes that release medications in reaction to temperature, pH, or other physiological cues have been made possible via nanotechnology. PEGylation and ligand conjugation are two surface changes that enhance targeting and lessen off-target effects. New techniques like ultrasound-mediated release and magnetic targeting provide precise, site-specific medication delivery. Furthermore, RNA-loaded liposomes and pro-drug liposomes provide accurate delivery with less systemic toxicity [45].

- Polymeric Nanoparticles

Biodegradable polymers like PLGA and PLA play a crucial role in bone-targeted drug delivery systems due to their controlled-release capabilities. Incorporating drug-loaded NPs into tissue-engineered scaffolds enhances therapeutic efficacy for bone repair. In one study, PLGA NPs were embedded into a chitosan–bioactive-glass (CH-BG) scaffold via lyophilization. Compared to NP-free scaffolds, NP-loaded scaffolds showed improved mechanical strength and slightly reduced swelling, without altering morphology. The system demonstrated promise for sustained drug release post-implantation. Additionally, tuning NP size may further optimize biodistribution and targeted delivery in clinically relevant applications [46].

PLGA, an FDA-approved polymer, effectively encapsulates and delivers drugs but shows limited compatibility with certain molecules due to hydrophilicity or charge repulsion. To overcome this, hybrid systems combining PLGA or polycaprolactone with positively charged chitosan have been explored to improve drug loading. However, conventional coupling methods often involve harsh conditions or yield unrefined products. This study evaluated two synthetic approaches for producing pure, tunable PLGA–chitosan hybrids, using NMR, FT-IR, and DSC for characterization. The resulting materials demonstrated improved compatibility with previously incompatible drugs, providing a promising platform for enhanced delivery [47]. Compared to synthetic polymers, natural polymers often have advantages such as biocompatibility, biodegradability, and biologically identifiable moieties that support cellular activities. A naturally occurring polymer made from chitin, chitosan, has recently attracted interest because of its stability, non-toxicity, biodegradability, biocompatibility, and sterilizability. Additional characteristics of chitosan include its speed of drug release, ease of modification, capacity to cross-link with other polymers, antibacterial qualities, gel formation, bio-adhesion, macrophage activation, immunostimulation, and gas permeability [48].

- Micelles

Amphiphilic block copolymers enhance micellar nanocarrier stability and drug loading by self-assembling hydrophilic–hydrophobic architecture. While hemiacetal ester-based polymers offer favorable degradation profiles for (immuno)drug delivery, they present difficulties due to their limited loading capacity and quick hydrolysis. To increase medication encapsulation and stabilize the hydrophobic micellar core, a study used a monomer containing phenyl. Covalent dye labeling was made possible by post-polymerization alterations using activated ester groups, which improved carrier functionality and offered insightful mechanistic information [49].

A study used two series of amphiphilic polymer micelles, PEG-b-PCLm and PEG-b-PCLm/TPGS, to create a complex drug delivery system that uses multi-arm amphiphilic block copolymers to increase cancer therapy efficacy. By using solvent dialysis, doxorubicin (DOX) was incorporated into the micelles. The micelles had a homogeneous shape, restricted size distribution, and excellent biocompatibility. Compared to free DOX, DOX-loaded micelles showed enhanced drug accumulation at tumor sites and greater anticancer effectiveness. Furthermore, drug-resistant MCF-7/ADR cells were efficiently suppressed by 4A-PEG47-b-PCL21/TPGS micelles. An inventive micelle formulation with exceptional serum stability and efficacy against drug resistance was revealed in this work, potentially leading to the treatment of cancer. It highlights cutting-edge methods for improving clinical translation and ensuring long-term safety and efficacy in vivo [50]. Amphiphilic copolymer micelles that respond to stimuli have attracted a lot of attention. Certain physical triggers, such as light, temperature, etc., might cause these micelles to react to chemical cues, such as redox potential and pH, as well as physiological elements (ATP, enzymes, etc.). The micelles’ features or attributes may change in response to various stimuli, enabling focused treatment and controlled medication release in malignancies. These stimuli-responsive tactics offer fresh approaches to enhancing tumor effectiveness and reducing adverse medication reactions [51].

### 4.2. Advanced Nanocarriers

- Dendrimers

The distinctive symmetrical branching structure of dendrimers, a family of three-dimensional, nanoscale hyperbranched polymers, sets them apart. The term ‘dendrimer’ comes from the Greek word ‘dendron’, meaning ‘tree’, reflecting their distinctive ‘tree-like’ branching structure. To produce a chemically well-defined product, dendrimers are often synthesized using an iterative coupling process that uses either a divergent or convergent approach [52,53,54]. Additionally, dendrimers with a large number of terminal groups have multifunctional properties that enable highly localized surface conjugation of different functional agents (e.g., targeting ligands) [55]. The flexibility and deformability of dendrimers, along with the high local concentration of ligands, provide a multivalent binding effect that greatly increases the tendency to bind to complementary receptors. Dendrimers’ unique internal structure creates internal pockets or holes that can enclose hydrophobic molecules, increasing their water solubility [56].

Dendrimer-based nanocarriers offer numerous structural advantages, making them exceptionally efficient for drug delivery applications. Their distinctive internal structure creates a void volume that enables the encapsulation of hydrophobic drugs, enhancing their solubility in water. Moreover, higher-generation dendrimers (G4 and beyond) provide enhanced drug loading capacity, owing to their greater internal volumes. The compact surface architecture further improves their encapsulation efficiency and drug retention, thereby reducing premature drug release [57,58].

Research indicates that over 70% of methotrexate (MTX) can be liberated from G5 PAMAM dendrimers within 2.5 h in phosphate-buffered saline (PBS), while negligible release transpires in water, suggesting that physiological buffer salts diminish dendrimer–drug interactions. Covalent conjugation of therapeutic agents to the various peripheral functional groups on dendrimers mitigates uncontrolled burst release. Stimuli-responsive drug release mechanisms, including acid-labile hydrazone, hydrolyzable ester, and reducible disulfide linkers, facilitate controlled drug release in response to particular physiological conditions, such as the low pH characteristic of tumor environments. Moreover, hybrid nanocarrier configurations, such as dendrimer-based micelles (LDBC-based micelles) and Janus dendrimersomes, provide superior drug encapsulation, extended release characteristics, and enhanced thermodynamic stability, thereby optimizing drug delivery efficacy [59,60]. For example, MTX’s attachment to folate-targeted PAMAM G5 dendrimers via an ester linker results in enhanced cytotoxicity against KB cells because lysosomal enzymes expedite the lysosomal hydrolysis process. In a mouse model of breast cancer, a PEGylated peptide dendrimer–doxorubicin (DOX) combination using an enzyme-responsive tetrapeptide linker demonstrated a 35% increase in tumor growth suppression in comparison to free DOX [61,62].

To improve stability and encapsulation efficiency, other dendrimer derivatives have also been produced, such as linear-dendritic block copolymers (LDBCs) and Janus dendrimers. Compared to linear polymeric micelles, PAMAM-based micelles with dendritic blocks showed noticeably better drug encapsulation. With critical micelle concentrations (CMC) one to two orders of magnitude lower than those of their linear counterparts, LDBC-based micelles showed improved thermodynamic stability and more controlled drug release. Dendrimersomes derived from amphiphilic Janus dendrimers exhibited negligible drug leakage at physiological pH, while facilitating increased drug release in acidic tumor-mimicking environments. These findings emphasize the promise of dendrimers as adaptable and highly customizable nanocarriers, while also highlighting the necessity to mitigate their toxicity and manufacturing challenges for clinical use [63,64,65].

Notwithstanding these benefits, dendrimer-based nanocarriers encounter considerable obstacles. Their extensively branched architecture and surface charge may induce cytotoxic effects, requiring meticulous surface modification to improve biocompatibility. Moreover, synthesizing high-generation dendrimers with meticulous control over size and functionalization remains technically intricate and costly. Encapsulated pharmaceuticals may experience premature burst release under physiological conditions, necessitating the use of supplementary functionalization strategies to enhance retention. Moreover, excessive drug conjugation can alter the material’s properties, leading to increased polydispersity or reduced solubility, which may compromise the efficacy of the nanocarrier [66]. Dendrimers possess distinctive structural benefits and diverse functionalization options, rendering them effective nanocarriers for drug delivery. Nonetheless, their clinical application is impeded by toxicity issues, synthesis difficulties, and potential drug release complications. Subsequent investigations should focus on developing safer dendrimer formulations that exhibit enhanced stability and controlled release mechanisms, thereby fully leveraging their capabilities in targeted drug delivery.

### 4.3. Mesoporous Silica Nanoparticles

**Figure 4:**
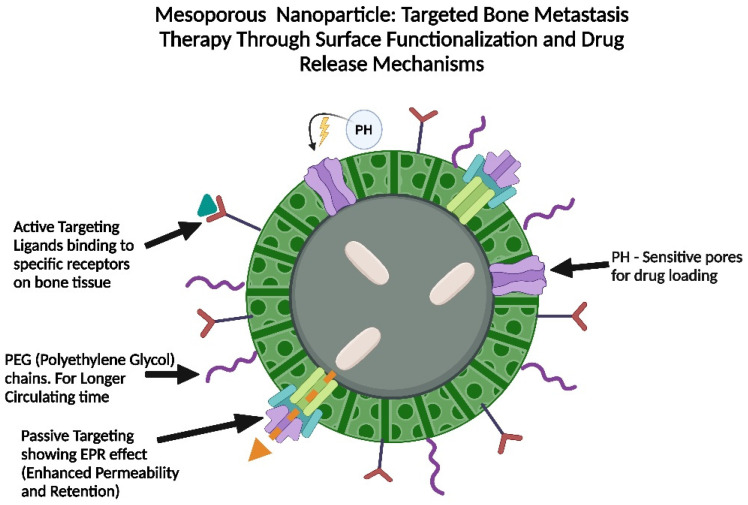
Design of mesoporous nanoparticles for targeted bone metastasis therapy. The figure shows a mesoporous nanoparticle featuring pH-sensitive pores for drug loading, PEG chains for enhanced circulation, and surface ligands for active targeting of bone tissue. It also details the EPR effect for passive targeting.

Mesoporous silica nanoparticles (MSNs) possess a highly organized porous architecture, extensive surface area, and adjustable dimensions, rendering them optimal for drug encapsulation and regulated release in treating bone metastasis. Their bifunctional surfaces facilitate selective modifications, permitting targeted delivery to bone tissue. Moreover, MSNs demonstrate excellent biocompatibility and biodegradability, guaranteeing secure and effective uses in therapy. Clotrimazole and paclitaxel, when incorporated into (MSNs), exhibited augment dissolution rates and heighten cytotoxicity against cancer cells. The spatial restriction within mesopores hinders drug crystallization, leading to enhanced absorption and improved therapeutic effectiveness. Moreover, the pore size of MSNs can be modified to regulate the kinetics of drug release (Figure 4). Research indicates that reduced pore size impedes drug diffusion, facilitating a prolonged release profile. The regulated release is essential for bone metastasis treatment, as it requires localized, sustained drug delivery to effectively target tumor cells while reducing systemic toxicity [67,68,69,70].

Through the use of a noncovalent post-modification technique, it is possible to create MSN-based drug delivery systems with enhanced therapeutic efficacy in a simple one-pot procedure by encasing tiny anticancer agents inside the unmodified mesopores and then blocking the drug-loaded pores with a stimuli-responsive polymer gatekeeper. Drug-loaded MSNs can be altered with the right targeting components, such as synthetic protein coronas or targeting ligands, for accurate delivery [71].

The release of drugs from (MSNs) can be meticulously regulated through diverse gatekeeper systems, including pH-sensitive and redox-sensitive mechanisms. Redox-sensitive systems utilize the intracellular glutathione concentration, which may attain levels of up to 10 mM, to initiate drug release. Disulfide bonds connecting the capping system to the MSN are diminished upon cytoplasmic entry, resulting in the regulated release of the cargo [72].

A study employed β-cyclodextrin covalently bonded to MSN through disulfide linkage, exhibiting effective doxorubicin cytotoxicity in lung adenocarcinoma cells. Cysteine-crosslinked polymers were used to block MSN pores, and the polymeric network broke down in a reducing environment to facilitate drug release [73,74].

Moreover, pH-sensitive methods have been developed to help to release medications in tumor microenvironments that are acidic. By combining an amide and a disulfide linker, Li et al. created a dual-responsive system that uses ammonium salt to block the pores of MSNs. During cellular absorption, the amide bond breaks down at a low pH, whereas the disulfide link is reduced in a glutathione-dependent way, allowing for targeted and regulated drug release. MSNs are positioned as a promising platform for targeted cancer treatment because of these sophisticated drug delivery systems, which increase therapeutic effectiveness while lowering off-target consequences [75].

Through active or passive targeting strategies, MSNs can accumulate within tumors. Because tumors often have abnormal vasculature and insufficient lymphatic drainage, the increased permeability and retention effect promote the passive accumulation of nanoparticles in tumor tissue. However, surface alterations, such as PEGylation, have a significant impact on this accumulation. PEGylation is widely used to prevent early clearance from the bloodstream by extending circulation duration and preventing detection by the mononuclear phagocyte system (MPS). Despite its benefits, PEGylation has been demonstrated to decrease cellular absorption in macrophages and cancer cells, which may restrict the effectiveness of nanoparticles in specific situations [76,77,78]. However, certain studies, including one by Zhu et al., have indicated that PEGylated MSNs exhibited enhanced uptake in cervical cancer cells relative to unmodified nanoparticles [79]. Furthermore, tumors exhibiting necrotic regions or inadequate blood supply may hinder passive targeting, as nanoparticles may not adequately access these areas due to heterogeneous blood flow and increased interstitial fluid pressure [80].

Moreover, investigations into the biodistribution of MSN revealed that, although nanoparticles predominantly concentrated in organs such as the liver and spleen, they were rapidly eliminated from these organs within a few weeks. PEGylation impeded the clearance of nanoparticles, with smaller particles exhibiting extended blood circulation. These findings indicate that PEGylation can enhance circulation time; however, the distribution of nanoparticles within tumors and other organs may be affected by particle size and surface modification [81,82].

### 4.4. Inorganic Nanoparticles (Metal/Metal Oxide)

Because of their special characteristics, gold NPs are useful in medication delivery, anticancer research, and antibacterial applications. Localized surface plasmon resonance (LSPR), improved biosensing, photothermal and photodynamic cancer treatments, targeted drug delivery, and bioimaging are some of their outstanding optical characteristics. Proteins, medications, and nucleic acids can be delivered effectively owing to the surface modification of gold nanoparticles.

In cancer research, gold NPs are effective in photothermal and photodynamic therapies, while their antimicrobial potential is demonstrated through the enhanced delivery of antigens, peptides, and antibiotics, promoting cell penetration and apoptosis. Compared to other metal nanoparticles, gold NPs offer lower toxicity, making them versatile tools in nanomedicine [83].

Nano-photosensitizer-mediated photothermal therapy inhibits tumor growth via various mechanisms, including thermal ablation and the activation of the antitumor immune response. A study designated gold (Au) nanoparticles as the primary photosensitizer. The photothermal conversion of gold is enhanced via a dopamine coating, to which the chemotherapy agent, doxorubicin (DOX), is conjugated. An acidic environment triggered the release of DOX from DOX-loaded, dopamine-modified gold nanoparticles (Au@PDA@DOX). The integration of chemotherapy and thermal ablation more effectively suppresses the proliferation, migration, and invasion of tumor cells, while also inducing apoptosis and necrosis in vitro. Furthermore, thermal ablation precipitates the immunogenic demise of prostate tumor cells (RM-1). The transformation of prostate tumors from a “cold” to a “hot” tumor exhibiting immunogenicity is noted. The combination effectively stimulates the antitumor immune response, suppresses tumor growth, and mitigates tumor-induced bone damage in vivo. Au@PDA@DOX nanoparticles are proposed as an innovative therapeutic approach for bone metastases in prostate tumors [84].

Although photothermal treatment (PTT) and medication delivery based on nanoparticles have shown promise for subcutaneous cancers, the complexity of the bone microenvironment and poor accessibility constrains their use in bone metastases. In order to improve the effectiveness of chemotherapy and PTT, a study presented a straightforward nanoplatform that combines gold nanorods (GNR) with thermosensitive liposomes (TSL). TSLs were engineered for physiological stability and heat-triggered drug release, then integrated with GNRs via incubation. In vitro, near-infrared (NIR) irradiation induced PTT, promoted drug release, and enhanced tumor cell sensitivity, reducing viability and migration. In a murine bone metastasis model, the nanoplatform enabled targeted drug/GNR delivery and localized release, preserving the bone structure, reducing pain, and extending survival by rebalancing osteoclast/osteoblast activity. This straightforward liposome–GNR system holds strong therapeutic potential for bone metastasis [42].

### 4.5. Iron Oxide Nanoparticles for Magnetic Targeting and MRI Contrast

Because of their special magnetic characteristics and biocompatibility, iron oxide nanoparticles (IONPs), especially superparamagnetic iron oxide nanoparticles (SPIONs), are useful for magnetic targeting and improving MRI contrast. They are widely employed in targeted therapeutics and diagnostics, especially in managing bone metastases.

SPIONs enhance MRI and magnetic targeting, improving diagnostic and therapeutic approaches through superior contrast effects and tunable properties. In the SPFeNOC system, FeO_4_nanoparticles target osteoclasts and metastatic cancer cells to improve MR imaging and aid in chemodynamic treatment (CDT). They are potential tools for controlling bone metastases because of their dual functioning, which enables accurate tumor localization and better therapy outcomes [85,86].

A study created a theranostic nanocarrier with folic acid-polyamidoamine (FA-PAMAM) dendrimers adorning a core of superparamagnetic iron oxide nanoparticles (SPION). To increase solubility and therapeutic efficacy, 3,4-difluorobenzylidene-curcumin (CDF), a hydrophobic anticancer drug, was co-encapsulated within the dendrimers. When compared to non-targeted versions, the targeted nanoparticles (SPIONs@FA-PAMAM-CDF) showed improved accumulation and anticancer activity in folate receptor-overexpressing ovarian (SKOV3) and cervical (HeLa) cancer cells, as well as superior magnetic resonance (MR) contrast. Their promise for concurrent cancer imaging and therapy was confirmed by this result, which was linked to multivalent folate receptor binding and the triggering of apoptosis by PTEN overexpression, caspase 3 activation, and NF-κB suppression [87].

Magnetic iron oxide (IO) nanoparticles are promising nanomaterials for in vitro and in vivo biomedical applications due to their prolonged blood retention, biodegradability, and low toxicity. Their large surface area allows functionalization with targeting ligands, such as antibodies, peptides, or small molecules, enhancing diagnostic imaging and therapeutic delivery.

IO nanoparticles exhibit strong paramagnetic properties, providing significant T2, T2*, and T1 contrast effects at low concentrations, making them effective in MRI for clinical oncology. However, challenges remain, including variable receptor expression in tumor cells, physiological barriers limiting nanoparticle access, and insufficient data on their intratumoral distribution and imaging efficiency in primary and metastatic tumors [88].

### 4.6. Hybrid Nanosystems

Inorganic–organic hybrid nanosystems have been developed over time for their adaptability and effectiveness in addressing challenges that nonhybridized counterparts cannot easily overcome. Presently, hybrid nanosystems are utilized for gene therapy, drug delivery, tissue regeneration, phototherapy, antibacterials, imaging probes, vaccines, biomolecule detection, and theranostics. Despite their diversity, these nanosystems can be categorized based on their fundamental inorganic or organic components, hybridization architecture, and auxiliary moieties [89]. Hybrid nanosystems combining organic and inorganic materials offer a promising approach for multimodal cancer therapy. While inorganic nanoparticles provide stability and multifunctionality, concerns about their biodegradability and biocompatibility remain. To address this, hollow mesoporous organosilica nanocapsules (HMONs) were developed with high dispersity and sizes below 50 nm. These HMONs incorporate a disulfide bond (-S-S-) that degrades in the reducing tumor microenvironment, enabling controlled drug release. Their small size promotes efficient tumor accumulation, enhancing chemotherapeutic efficacy. This hybrid design offers improved biodegradability, tumor responsiveness, and high drug-loading efficiency, making them highly effective for multimodal cancer therapy [90].

Different therapeutic compounds can be directed to different organs, including the liver, spleen, kidney, bone, and brain, by altering the ligand on the inorganic nanoparticle’s surface. However, there is little research on directing therapeutics to bone marrow cells, even though other focused nanomedicines have a wealth of data. As a result, the use of treatments for bone-related conditions, such as bone metastases, leads to a number of problems, such as severe systemic toxicity and insufficient effectiveness. A glutamate ligand, which is said to have a high affinity for bone cell NMDA receptors, has been successfully used in a study to functionalize a model inorganic nanoparticle (Fe_2_O_3_). The nano-hybrid was characterized using spectroscopic analyses. The ability of the Fe_2_O_3_ nanoparticle to generate photo-induced reactive oxygen species (ROS) suggests that it may be used therapeutically to treat bone metastases. Additionally, when exposed to X-ray radiation, the nanoparticle can release more reactive oxygen species, which could provide a new treatment option for cancer and bone metastases [91].

An inorganic–organic hybrid nanocomposite comprising polydopamine (PDA) and zirconium dioxide (ZrO_2_) was created for chemo-photothermal treatment and multimodal imaging. T-weighted magnetic resonance imaging (MRI) is made possible by the integration of Mn2+ ions, while computed tomography (CT) imaging is made possible by ZrO_2_, both of which have strong diagnostic potential. Under near-infrared (NIR) irradiation in acidic tumor environments, the nanoplatform effectively delivers doxorubicin, with PDA enhancing biocompatibility, photothermal conversion, and controlled drug release. The promise of multimodal cancer treatment is underscored by this composite system, which integrates chemotherapy, photothermal therapy, and CT/MR imaging within a single platform to provide precise cancer diagnosis and therapy [92].

### 4.7. Surface Modifications and Targeting Ligands

Surface modifications and targeting ligands are crucial for directing nanocarriers to bone metastases, thereby enhancing drug retention and efficacy. While bone-targeting agents like peptides (e.g., D10) and bisphosphonates show potential, their use is limited by short circulation and low bone accumulation. To address this, researchers conjugated D10 peptides to an Fc-mCherry fusion protein, significantly enhancing circulation (~80-fold) and bone uptake (13.6% in the femur, 11.4% in vertebrae at 24 h). An optimized anti-sclerostin-D10 antibody further improved retention (up to 20.9% in the femur, 19.5% in vertebrae at 7 days) and efficacy in bone loss models. These results underscore the value of combining antibodies, peptides, and bisphosphonates to enhance targeting, reduce dosing, and maintain therapeutic outcomes in bone metastasis treatment [93].

Bone-targeting nanoparticles that are coated with ligands such as bisphosphonates exhibit a strong affinity for bone; however, their firm binding to calcium can impede the effective targeting of tumors. To resolve this issue, researchers created a detachable nanocarrier that delivers Bortezomib (BTZ), a proteasome inhibitor, by utilizing ZIF-8 and an MMP-sensitive, D8-peptide-modified hyaluronic acid (HA). The transport of drugs from bone to metastatic tumors is improved by these nanocarriers, which exhibit acid-sensitive drug release, D8-peptide-mediated bone targeting, and matrix metalloproteinase (MMP)-responsive detachment. HA targeting improves nanoparticle uptake and cytotoxicity by interacting with CD44-overexpressing tumor cells, boosting therapeutic efficacy. This cascade-targeting strategy demonstrates the potential of surface-modified nanoparticles with versatile ligands for effective bone metastasis treatment [94]. Bone-targeting ligands, including bisphosphonates, bone-homing peptides, antibodies, and aptamers (e.g., targeting HER2 and PSMA), are employed in nanocarriers to facilitate effective drug delivery in bone metastasis, thereby augmenting the pharmacological efficacy of antitumor and diagnostic agents [21,22]. The latest developments in bone-targeting drug delivery systems have investigated bisphosphonate-functionalized nanocarriers to improve therapeutic efficacy in bone metastases. Alendronate (ALN)-anchored biodegradable polymeric micelles were created by researchers for the purpose of treating bone metastases. These micelles enhance bone protection by modulating osteoclast activity while simultaneously delivering sustained docetaxel (DTX) release, improved cytotoxicity, and superior pharmacokinetics compared to conventional formulations. DTX-loaded ALN micelles significantly reduced tumor burden and enhanced survival rates in animal models of breast cancer bone metastasis. These findings highlight the potential of bisphosphonate-modified nanocarriers for enhancing drug retention and therapeutic efficacy in bone-targeted therapy [95].

## 5. Mechanisms of Bone-Targeted Delivery

The bone microenvironment provides distinct signals that affect cancer proliferation, immunogenicity, and metastasis. Conventional cancer treatments exhibit restricted efficacy owing to off-target effects and inadequate distribution in bone tissue. Consequently, there is a significant demand for therapies that exhibit enhanced specificity and efficacy in treating bone tumors. A highly promising strategy involves the precise delivery of pharmaceutical agents to the bone cancer site by incorporating bone-targeting moieties, such as bisphosphonates or oligopeptides. These moieties exhibit strong affinities for the bone hydroxyapatite matrix, a structure unique to skeletal tissue, and can improve the targeting capability and effectiveness of anticancer drugs in the treatment of bone tumors [96].

Biomineralized metal–organic framework (MOF) nanoparticles encapsulating protein toxins were developed for the active targeting of bone metastases. These dual-targeting nanocarriers bind both bone tissue and CD44 receptors, which are overexpressed in tumor cells, enabling the precise delivery of protein therapeutics. Integration with a RANKL antibody further enhanced the efficacy of ribosome-inactivating proteins (RIP), disrupting tumor–bone interactions, reducing skeletal-related events, and improving therapeutic outcomes. This approach highlights the potential of ligand–receptor strategies to enhance targeting accuracy and treatment effectiveness in bone metastasis [97].

### 5.1. Stimuli-Responsive Systems

The bone metastatic niches frequently display an acidic microenvironment resulting from enhanced osteoclastic activity and tumor metabolism. pH-sensitive nanoparticles are engineered to utilize this property by discharging their payload in response to the decreased pH levels [98]. The action of plasma membrane proton pumps and increased glycolysis cause the pH of the tumor microenvironment (TME) to be somewhat lower than that of healthy cells. The extracellular pH in most tumors is between 6.5 and 7.2, but the pH inside lysosomes is much lower, ranging from 5.0 to 5.5 [30,99]. The acidic characteristics of TME can be used to implement pH-sensitive nanoparticle drug delivery systems that selectively release medicines at low pH values [30]. pH-responsive nanoparticles are a distinctive category of nanoparticles that demonstrate significant sensitivity to variations in environmental pH [100,101]. The reactivity of these nanoparticles is attributed to their unique structure and composition, typically involving components such as polyamides, polyacrylic acid, and silicates [102].

pH-responsive nanoparticles typically exist in two states: a resting state and an activated state [103]. Nanoparticles frequently maintain an at-rest state in neutral or nearly neutral environments, characterized by decreased volumes and surface charges. However, the nanoparticles are activated by changes in the pH of the environment, which causes changes in their chemical and physical properties. pH-responsive nanoparticles have unique characteristics in both acidic and alkaline environments. These nanoparticles preferentially absorb protons in acidic environments, creating a positive charge that repels other particles and eventually causes them to accumulate. Nanoparticles release protons and pick up a negative charge in alkaline environments, attracting particles and promoting their dispersion. pH-responsive nanoparticles’ special properties allow them to play crucial roles in a variety of fields, such as biomedicine, environmental monitoring, and the creation of intelligent materials. In medication delivery, the pH responsiveness of nanoparticles can be utilized to facilitate targeted drug release, hence improving therapeutic efficacy and minimizing side effects [30,104]. pH-responsive nanoparticles can be employed in environmental monitoring to detect pH fluctuations in aquatic systems, yielding precise data for environmental assessment [105]. Moreover, by modifying the pH sensitivity of nanoparticles, advanced materials like intelligent coatings and sensors can be created [106]. Zhao et al. engineered redox and pH dual-sensitive, bone-targeting nanoparticles that release therapeutic drugs exclusively in the acidic and reductive conditions characteristic of bone metastases, hence improving site-specific delivery [98]. Yang et al. used ZSM-5 zeolite as a carrier for the anticancer medication DOX to create ZSM-5/CS/DOX core–shell nanodisks, utilizing chitosan (CS) as the shell material [107]. It was discovered that ZSM-5-zeolite-derived components improved osteoblast differentiation by inhibiting NF-κB activation, as documented by Zhou et al. [108]. The ZSM-5/CS/DOX core–shell nanodisks exhibited a diameter of 100 nm, a pore size of 3.75 nm, and a remarkable drug loading capacity of 97.7%. The positively charged surface of CS imparted pH-responsive behavior to the ZSM-5/CS/DOX core–shell nanodisks. At pH 6, the ZSM-5/CS/DOX core–shell nanodisks released 58.7% of DOX, demonstrating a pH-dependent drug release characteristic [30].

Zhu et al. proposed a technique that integrates bone targeting with pH-responsive medication release for anti-metastatic therapy. Zhu et al. showed that prodrug micelles are effective at guiding drug delivery to bone metastatic tumor tissues, and the synthesized micelle ALN-NP showed improved therapeutic efficacy and reduced systemic toxicity when compared to free medications or control micelles, resulting in improved therapeutic outcomes and fewer adverse effects [109]. In bone reconstruction contexts, the presence of particular enzymes implicated in the bone remodeling process, including cathepsin K, various matrix metalloproteinases (e.g., MMPs -2, -9, -13, -14, and -16), and vacuolar H+ ATPase (an osteoclastic enzyme featuring a distinctive 116-kD subunit that may be utilized for targeted applications) serve as significant triggers for utilization. Nanoparticles designed with MMP-sensitive linkers can maintain stability in circulation while releasing their therapeutic payload upon interaction with these enzymes at the metastatic location. This technique enables accurate medication release in response to the enzymatic activity linked to bone metastases [110]. The most advanced bone-oriented stimulus-responsive designs are collated in Table 2, highlighting how dual pH- and redox-gated carriers, mild-hyperthermia liposomes, magnetically heated vesicles, and focused-ultrasound liposomes translate these principles in vivo.

### 5.2. Thermal-, Magnetic-, or Ultrasound-Triggered Release

In recent years, stimuli-responsive nanocarriers have been explored as a method to overcome the natural burst release profile of nano-formulated therapies and the residual release during parenteral delivery. To attain this control, these designated smart delivery systems can be meticulously customized to react to external, internal, or physiological triggering factors to facilitate the precise release of bioactive compounds. Stimuli-responsive nanocarriers have been designed to react to triggers including pH gradients, redox conditions, light, ultrasound, magnetic fields, temperature, and enzymes [110].

The low critical solution temperature (LCST) of various substances utilized in nanocarrier assembly is a crucial parameter for customizing the temperature-mediated release profile of these delivery systems. In light of their innate biological characteristics, healthy people have thermoregulatory systems that maintain a constant body temperature over time. Nevertheless, certain pathophysiological conditions, such as inflammation and tumors, exhibit elevated temperatures compared to healthy tissues [110,111].

The heat disparity between cancerous and normal tissues has been thoroughly investigated for the development of thermo-responsive nanocarriers as therapies for cancer, as illustrated below. Nevertheless, due to the heterogeneous response of tumors to localized hyperthermia, the efficacy of this targeted administration could be enhanced by externally elevating the temperature at the tumor site via ultrasound, alternating magnetic fields, or temperature-regulated water sacks [110].

**Figure 5:**
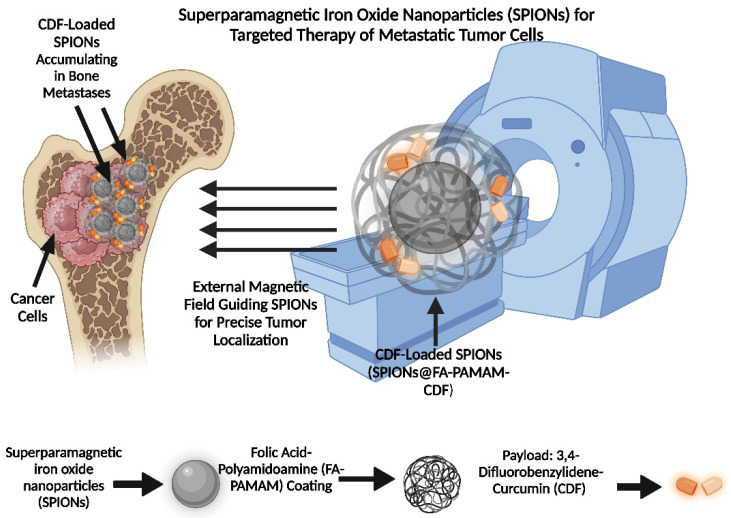
Superparamagnetic iron oxide nanoparticles (SPIONs) for targeted therapy of metastatic tumor cells. The diagram displays the structure of CDF-loaded SPIONs, featuring an external magnetic field application for tumor localization, and details components like the superparamagnetic iron oxide core, folic-acid-polyamidoamine coating, and 3,4-difluorobenzylidene-curcumin payload.

Superparamagnetic iron oxide nanoparticles (SPIONs) can be guided to the bone metastasis location via an external magnetic field (Figure 5). The superparamagnetic IONPs are independently stabilized with palmityl-nitroDOPA integrated into the lipid membrane. Localized alternating magnetic fields can be employed to regulate the amount and timing of cargo released from vesicles by locally warming the membrane, thereby altering its permeability without significantly impacting the surrounding environment [110,112].

Ultrasound waves can infiltrate deep tissues and generate mechanical vibrations or localized heating; moreover, this non-invasive method facilitates spatial and temporal regulation of drug release, rendering it a significant asset in the management of bone metastases [110].

In the specific instance of bone tissues, Staruch and colleagues successfully accomplished MRI-guided drug deposition in bone by the delivery of thermo-responsive liposomes containing DOX (ThermoDox^®^) [113]. The application of focused ultrasonic heating following liposomal injection in New Zealand white rabbits led to an 8.2- and 16.8-fold augmentation of DOX content in bone marrow and surrounding muscle tissues, respectively, relative to non-heated tissues [110].

## 6. Therapeutic Cargoes and Combination Strategies

### 6.1. Chemotherapeutic Agents

Combination therapy has reduced side effects and increased antitumor activity. Drug delivery systems based on nanoparticles offer a promising strategy for combination cancer treatment by encapsulating many chemotherapeutic drugs at once. However, inadequate drug release from nanomedicines in cancer cells could reduce their anticancer effectiveness. mPEG-PαLA is a dual-responsive nanocarrier that can simultaneously encapsulate PTX and DOX and self-assemble into micelles in aquatic settings. K7 osteosarcoma cells were able to internalize this dual-drug-loaded micellar nanoparticle, which showed efficient drug release by a reduction/pH dual response. In a murine K7 osteosarcoma model, the dual-drug-loaded mPEG-PαLA micelles showed notable synergistic anticancer effects both in vitro and in vivo. This was ascribed to the improved biodistribution of the nanoparticles and the synergy between PTX and DOX. The substantial potential of the mPEG-PαLA copolymer as a dual-responsive nanocarrier for the co-delivery of anticancer drugs in the treatment of osteosarcoma is demonstrated in this study [114].

### 6.2. Dosing Strategies and Synergy with Bone Microenvironment

Bone metastases are common in advanced-stage malignancies, leading to significant morbidity and mortality. While the overexpression of RANKL in the bone microenvironment promotes metastatic progression and bone resorption, the poor biodistribution of anticancer drugs to bone restricts the efficiency of chemotherapy. Researchers tested a combination therapy using docetaxel-loaded nanoparticles (TXT-NPs) for targeted bone delivery and the monoclonal antibody denosumab (DNmb), which inhibits RANKL. In a prostate-cancer-induced osteolytic bone metastasis model, TXT-NPs achieved nearly three-fold higher drug concentrations in metastatic bone compared to standard TXT-CrEL. Combined TXT-NPs + DNmb treatment effectively suppressed tumor progression, induced early tumor regression, and improved survival without causing weight loss or relapse post-treatment. Micro-CT and histochemical analysis confirmed preserved bone structure, mineral content, and normal osteoblast/osteoclast activity, unlike monotherapies, which led to bone loss and tumor relapse. These findings demonstrate the promise of combining TXT-NPs and DNmb to effectively disrupt cancer–bone-cell interactions and enhance bone metastasis treatment [115].

## 7. Biologics and Gene Therapy

CRISPR/Cas9 organic nano-delivery systems must overcome various biological barriers to achieve effective tumor targeting and gene editing. Following injection, the endothelial barriers, the mononuclear phagocyte system, and the clearance mechanisms that impact circulation and biodistribution pose difficulties for these systems. Minimizing phagocyte absorption, decreasing interactions with blood components, and extending circulation are essential. Through EPR, nanocarriers build up in tumors and can be actively targeted by particular ligands. Membrane fusion or receptor-mediated endocytosis are the two ways that cells are taken up. After internalization, avoiding endosomal/lysosomal degradation is vital for getting the CRISPR/Cas system into the nucleus. Triggered release mechanisms can enhance delivery precision. The efficient clearance of nanoparticles and their byproducts is essential for reducing toxicity. Understanding how nanocarriers navigate biological barriers, target tumors, achieve cellular uptake, and facilitate controlled release is vital for optimizing delivery systems for effective cancer therapy [116].

RNA-based therapies hold promise for bone regeneration by promoting osteogenesis when delivered via scaffolds. Lipid and polymer nanoparticles are key RNA carriers, while hydrogels and collagen are commonly used for bone targeting. Despite encouraging results, clinical translation remains limited due to the complexity of bone healing, diverse injury types, and the influence of factors like stiffness and pH on RNA release and transfection. Although RNA can be released for over 50 days in vitro, sustained in vivo transfection remains a challenge. Scaffold stiffness impacts MSC differentiation, but its role in RNA delivery is not fully understood. Advancements such as 3D printing and nanocarriers (e.g., AuNPs) may improve delivery efficiency, offering potential for more effective and scalable RNA-based bone therapies [117].

### 7.1. The Role of Immunotherapy

Since the immune system in the tumor microenvironment has a major impact on the development of bone metastases, immunotherapy holds hope for treating bone metastases that were previously thought to be incurable. Researchers are investigating novel strategies that specifically target immune cells within bone metastases. To find out which patient cohorts benefit the most, researchers are now combining immunotherapy with other treatments like chemotherapy, radiation, and targeted medications [118]. This multifaceted strategy seeks to enhance early detection and maybe halt cancer progression. Nonetheless, bone metastases continue to pose a considerable issue. Research conducted by Angel Qin indicates that patients with bone metastases undergoing immune checkpoint inhibitor (ICI) therapy may experience reduced survival durations and fewer favorable responses compared to those without bone involvement, underscoring the necessity for additional investigation. Moreover, individuals with bone metastases frequently exhibit resistance to immune checkpoint inhibitors (ICIs) and generally experience poorer outcomes. Research on the impact of bone metastases on immunotherapy effectiveness is continuous, and ongoing clinical trials aim to clarify this relationship [119].

### 7.2. Combination Therapies

A study presents an overview of research findings that elucidate nanotechnology-assisted methods being employed or investigated to combat cancer spread. We emphasize the significant advantages that nanotechnology-assisted methods can provide, either independently or in conjunction with established conventional procedures, to enhance the management of the intricate and formidable metastatic disease. The distinctive methodology in the discourse herein is to correlate particular nanotechnology tactics with the intricate biology of various phases of the metastatic cascade. The integration of nanotechnology has greatly expanded many approaches for addressing cancer metastases, which range from diagnostics to prevention and treatment at several phases of the metastatic cascade. These developments reveal the great possibilities of nanotechnology in preventing metastases and emphasize its many uses in targeting particular components of the metastatic process [120].

Nanotechnology offers promising strategies to disrupt the metastatic cascade and inhibit cancer progression. While long-term safety remains under study, current data support its potential for targeted, effective metastasis treatment. Nanomaterials enhance cancer cell identification and combat metastasis by inducing apoptosis, targeting CSCs and CTCs, disrupting pre-metastatic niches, inhibiting EMT, modulating the tumor microenvironment, and boosting immune responses. They also improve conventional therapies via synergistic effects, enabling controlled drug delivery and enhanced cellular uptake. Targeting EMT and angiogenesis may further strengthen anti-metastatic outcomes, positioning nanoparticles as powerful tools for future cancer therapy and nanotheranostics [120].

Numerous studies have employed nanotechnology to directly target tumor cells or TME at the site of metastasis to reduce it. Sun et al. [121] conjugated zoledronic acid (ZOL), a third-generation nitrogen-containing bisphosphonate, onto mesoporous silica nanoparticle gold-coated nanorods (Au@MSNs-Z) as a combination treatment for breast cancer bone metastases. ZOL has a strong affinity for bone and can block farnesyl pyrophosphate synthase (which kills osteoclasts), lower VEGF, accelerate tumor cell death, and drive NPs to bone metastases. Au@MSNs-ZOL outperformed unmodified NPs in bone metastases photothermal therapy using laser light following intravenous administration to the metastatic site. Furthermore, in a mouse model of intraosseous injection of MDA-MB231 breast cancer cells, Au@MSNs-ZOL + laser effectively decreased bone metastases and pain [121]. Alendronate was coated onto a polyamidoamine (PAMAM) dendrimer in order to administer docetaxel that targets bone metastases. In vitro and in vivo, this combined treatment decreased the amount of lung cancer bone metastases and the mice’s pain response [122]. Small-molecule transcription factor loading A Gli2 inhibitor, alendronate-modified NPs lowered the tumor-associated bone lesion area and raised bone volume fraction in the mice’s tibiae. By decreasing NP blood circulation time and balancing bone-binding, this medication adjustment decreased the formulation’s nonphagocytic phagocytosis by macrophages. To get around bisphosphonates’ preference for bone tissue over tumor cells, folic acid was linked onto polylactic-co-glycolic acid (PLGA) NPs loaded with alendronate-modified paclitaxel (PTX) for dual bone/tumor metastasis-targeted treatment [123]. In vitro, NPs modified with folic acid enhanced the uptake of breast cancer cells. Dual targeting to increase tumor cellular uptake is helpful for treating bone metastases, as demonstrated by the prolonged survival time and decreased tumor growth rate of PTX-loaded NPs with the folic acid modification in vivo compared to those without. Layered double hydroxides (LDH) are multifunctional because they can be linked to a homing protein to deliver the medication to the metastatic location. This technique can transport the medication to the nucleus while shielding it from endonucleases [124,125].

### 7.3. Synergistic Effects to Overcome Resistance and Reduce Dosing

Medicine resistance brought on by extended chemotherapy poses a significant problem, since it increases anti-apoptotic proteins (such as Bcl-2, IAP, and Akt) and efflux transporters like P-gp, decreasing the effectiveness of the medicine and requiring greater dosages with more adverse effects. Although there are inhibitors that target these proteins, their toxicity limits their application. Drug delivery methods based on nanostructures have been investigated as a solution. Nanoparticles reduce off-target toxicity, increase medication solubility and stability, and improve drug accumulation at tumor locations through the EPR effect. This method presents a viable way to overcome resistance while lowering systemic adverse effects [126].

These nanoparticles continue to exhibit challenges, including inadequate tumor accumulation rates and inherent toxicity. Nonetheless, nanoparticle-based anticancer agents, such as Doxil^®^, are currently employed in clinical environments, evidencing their recognized effectiveness. Additionally, these nanocarriers can simultaneously carry anticancer drugs and drug resistance inhibitors, preventing the upregulation of proteins that cause resistance in real time and maximizing therapeutic effectiveness. Consequently, there is considerable potential for administering drug resistance inhibitors through nanoparticles, and the studies discussed in this review will probably mark important turning points in the development of nanoparticle-mediated delivery systems for resistance inhibitors [126].

## 8. Preclinical and Clinical Perspectives

### 8.1. In Vitro and In Vivo Models

Reliable in vitro models are essential for developing effective bone metastasis treatments, as traditional 2D models fail to predict clinical efficacy. Researchers have created a 3D co-culture model using spheroids to study the interactions between breast or prostate cancer cells and human bone marrow stromal cells, while also assessing cisplatin’s efficacy. Bone metastatic spheroids (BMSs) formed within 24 h, with breast and prostate cancer BMSs differing in size and circularity over time. Pre-labeling cells enabled imaging and analysis of cellular dynamics for up to 7 days. Cancer cells in BMSs showed increased proliferation and reduced sensitivity to cisplatin compared to those observed in 2D cultures. This adaptable 3D model offers a promising platform for screening new bone metastasis therapies [127].

Researchers have developed a 3D tissue-engineered bone model to study breast cancer bone metastasis, replicating bone remodeling dysregulation and drug responses. The model includes bone tissue from human mesenchymal stem cells, breast cancer cells (MDA-MB-231, MCF-7), and human osteoclasts to simulate the tumor–bone microenvironment. Both cancer cell lines induced bone loss, with MDA-MB-231 cells causing greater resorption. Drug testing showed that denosumab and zoledronic acid reduced bone loss, while zoledronic acid also inhibited cancer growth and invasion. iPTH therapy decreased bone resorption and cancer infiltration. This 3D model offers a cost-effective platform for drug screening and identifying new treatments for bone metastasis [128].

To investigate cancer cell dissemination, osteomimicry, and bone metastasis, researchers created in vitro and in vivo models that replicate primary prostate tumors and metastatic bone tissue. Utilizing 3D organoid cultures, adjustable matrix mechanics, and bone-derived growth factors, they investigated the impact of the bone microenvironment on the progression of prostate cancer (PCa). Their results indicated that bone-matrix-derived soluble factors (BMSFs) inhibited PCa cell proliferation but facilitated osteomimicry, whereas bone-marrow-derived stromal cells promoted tumor growth and migration. These models offer valuable platforms for the investigation of tumor progression, the testing of therapies, and the comprehension of the process by which the bone microenvironment promotes PCa metastasis, thereby facilitating the development of targeted treatment strategies [129].

Calcium-based nanoparticle systems have been designed to regulate calcium metabolism and the bone microenvironment in order to impede disease progression in bone metastases. These nanoparticles can affect bone remodeling processes, potentially interrupting the detrimental cycle of bone degradation and tumor proliferation. Preclinical assessments indicate that these nanoparticles can significantly modify the bone microenvironment, consequently diminishing tumor burden in bone tissues [130].

Nanotechnology-driven drug delivery systems have been investigated for the treatment of metastatic osteosarcoma. These systems aim to enhance drug bioavailability and reduce systemic toxicity. Preclinical studies have shown that nanoscale drug delivery technologies can efficiently transport therapeutic agents to metastatic locations, enhancing treatment efficacy and reducing adverse effects [131]. Nanostructured biomaterials have been employed to develop in vitro models that replicate the bone metastatic microenvironment. These models facilitate the evaluation of potential therapeutic interventions by allowing the examination of cancer cell behavior in a regulated environment. These nanocomposite scaffolds provide substantial platforms for the preclinical evaluation of novel therapies that are designed to address bone metastases by meticulously replicating the bone microenvironment [132].

### 8.2. Representative Preclinical Studies

Researchers have developed bone-targeting alendronate (ALN)-anchored biodegradable polymeric micelles for the targeted treatment of metastatic breast cancer in the bone. These micelles demonstrated a capacity to protect bone by modulating osteoclast activity. The encapsulation of DTX, a chemotherapeutic agent, within ALN-modified micelles led to prolonged release, increased cytotoxicity, and enhanced pharmacokinetics. In a syngeneic animal model of advanced disseminated breast cancer bone metastasis, treatment with these targeted DTX-loaded micelles reduced tumorigenesis and markedly enhanced animal lifespan relative to the conventional formulation [95].

## 9. Clinical Trials and Ongoing Studies

Until February 2025, the utilization of nano-drug delivery systems for bone metastases was almost exclusively in the preclinical research stage, with minimal advancement into clinical trials. A thorough examination of clinical trial databases and the latest research reveals a notable lack of Phase I–III clinical trials focusing on nano-drug delivery technologies for bone metastases. Nearly all of the progress in this domain has been recorded at the preclinical stage, where diverse nanoparticle-based approaches are investigated to enhance the delivery of medicines to bone metastatic sites. For example, a study has concentrated on calcium-based nanoparticle systems that regulate calcium metabolism and, in turn, the bone microenvironment to prevent disease progression, including cancer [130]. Moreover, investigations have concentrated on nanoparticles engineered to specifically target bone metastases in breast cancer, as the integration of targeted medicines with nanoparticles improves the precise delivery of therapeutic molecules to metastatic lesions, offering numerous benefits for therapy [12]. Although these are encouraging advancements, the translation into clinical studies has been constrained.

### Regulatory Status, Safety Profiles, and Reported Efficacy

A shortage of clinical trials in this area has led to a deficiency in regulatory approvals for nano-drug delivery methods targeting bone metastases. As a result, extensive safety profiles and efficacy data from human studies are currently unavailable. The U.S. Food and Drug Administration (FDA) has developed legislative rules and regulations for nanomedicine products [133]. Nonetheless, without clinical trial data, these nano-drug delivery systems for bone metastases have not progressed to the regulatory evaluation or authorization phase.

In summary, whereas preclinical investigations of nano-drug delivery methods for bone metastases exhibit considerable potential, the shift to clinical implementation remains pending. Current and upcoming clinical trials are crucial for assessing the safety and effectiveness of these novel methodologies, which may enhance therapy results for patients with bone metastatic cancer.

## 10. Challenges in Translation and Regulatory Considerations

### 10.1. Manufacturing and Scalability

Before being approved for sale, new nanoparticle treatments must pass several tests. This includes designing a consistent manufacturing process, creating a nanostructure with appropriate components and properties, choosing orthogonal analytical techniques for accurate characterization, achieving a favorable pharmacological and toxicity profile, and proving safety and effectiveness in clinical trials. The inherent complexity and multifaceted nature of nanomedicines introduce many additional variables that may significantly increase the difficulty of managing processes and predicting behavior within a biological system, even though these challenges are conceptually similar to those faced by any novel pharmaceutical. When generic nanomedicines apply for approval from health authorities based on claims of equivalency to the original drug, additional regulatory and developmental variables come into play [134]. 

Nanomedicine development will incorporate personalized medicine to determine which patients will most likely benefit, particularly in oncology, where it can expedite approval and streamline studies. Utilizing biological pathways could increase the effectiveness of nanomedicine, as tumors use them to proliferate. To keep up with the quick developments in nanomedicine, regulators must set rules and regulations [134].

### 10.2. Stability During Storage and Distribution

Nanocarrier stability is crucial for effective drug delivery, as issues such as phospholipid oxidation, reduced zeta potential, improper charge distribution, and Ostwald ripening can lead to drug leakage and reduced encapsulation efficiency. Self-assembled micelles and nanoemulsions exhibit varying stabilities; in addition, the low critical solution temperature (LCST) influences their thermal stability, with drug release typically occurring above the LCST. Nanoemulsions are particularly prone to degradation over time. Nanocarrier suspensions face stability challenges, with size deviations indicating dissociation or aggregation and turbidity changes suggesting instability. Exposure to biological matrices can alter their physicochemical properties, often detectable via spectroscopy, chromatography, microscopy, and Förster resonance energy transfer (FRET). However, FRET analysis can be affected by interactions with column beads. Studies show polyethylene glycol–polyvinyl pyrrolidone micelles have lower serum stability than polyester-based micelles [135].

## 11. Safety and Toxicological Issues

By enabling cutting-edge therapeutic, diagnostic, and sensing techniques, nanomaterials (NMs) have revolutionized many aspects of medicine. Applications of key categories of nanomaterials, such as polymers, metal/metal oxides, carbon, liposomes, and multi-scale macro/nano bulk materials, have grown significantly due to advancements in processing and production. Concerns regarding the general biocompatibility and nanotoxicity of nanomaterials have also surfaced at the same time. These include possible negative consequences for patients, as well as those exposed at work during production [136].

### Need for Standardized Toxicity Assessment Protocols

Nanotoxicology research and nanosafety research are closely related to nanotechnology research. These allied professions’ main goals are to investigate nanomaterials’ toxicity and work to improve human quality of life. Continuous improvements in disciplines like medicine can benefit human life and health in several ways. However, various sectors use nanoparticles, which are subsequently included in many products. Nanomaterials may induce several potential toxicological and harmful effects. Furthermore, they can adversely impact numerous ecosystems. The toxicological hazards associated with nanomaterials must be assessed to identify the potential risks of adverse effects on human health and the environment. Recent research publications suggest that widely employed methodologies have enabled the development of more effective nanomaterials while minimizing the associated risks of chemicals. In nanomedicine, the importance of nanotoxicology is crucial in preventing the toxicity of drug nanocarriers [137].

## 12. Regulatory Framework

### Gaps in the Regulation That May Hamper Clinical Translation

Despite promising preclinical results, clinical translation of nanomedicines remains slow, biased, and often unsuccessful. Challenges include inadequate protocols, inconsistent material characterization, poor statistical analysis, diverse models, limited data sharing, and flawed study designs. Only 20–25% of preclinical biology research progresses to oncology, with success rates dropping from 94% in Phase I trials to 48% in Phase II and 14% in Phase III.

Because even little changes can significantly influence biodistribution and tolerance, successful clinical translation necessitates a thorough assessment of biocompatibility, immunotoxicity, pharmacotoxicology, and manufacturing procedures. The crucial tests include hemolysis, complement activation, cytokine release, opsonization, phagocytosis, pharmacokinetic (ADME) studies, dosing regimen, maximum tolerated dose, delivery route, and therapeutic index. Multidisciplinary collaboration integrating material science, accurate disease models, and regulatory alignment is critical for generating reliable data to advance nanomedicines from research to market approval, addressing global healthcare challenges [138].

## 13. Future Outlook: Emerging Trends, Challenges, and Innovations

### 13.1. Multifunctional “Smart” Nanocarriers

Recent advancements in nanoparticle-based drug delivery and diagnostics offer promising solutions to challenges in cancer treatment, such as drug resistance, side effects, and tumor microenvironment barriers. Polymeric nanoparticles, valued for their biocompatibility, biodegradability, controlled release, and targeting capabilities, are ideal for theranostic platforms that combine imaging and therapy. Their versatility allows for various designs, including polymer conjugates, dendrimers, micelles, and nanospheres, suitable for targeted gene and drug delivery. They can also incorporate imaging agents for use in radiotherapy, photodynamic therapy (PDT), and photothermal therapy (PTT). Despite progress, most studies remain at the in vitro stage, highlighting the need for strategies to advance these nanosystems to clinical use. Future efforts should focus on integrating multimodal treatments and precision medicine to enhance diagnostics, overcome tumor resistance, and improve personalized cancer therapy [139].

### 13.2. Advanced Manufacturing Techniques

The production of nanoparticles on microfluidic devices offers superior reproducibility and precise control compared to bulk synthesis. Although numerous platforms exist to produce (NPs) with regulated physicochemical features, these platforms frequently function within a limited spectrum of predetermined flow rates. The flow rate limitation limits both downscaling for exploratory research applications and upscaling for industrial production. They present a universal flow rate platform that maintains optimal mixing efficiency while operating over a wide range of flow rates (0.1–75 mL/min) for both small-scale exploratory research and industrial-scale nanoparticle manufacturing. Using coaxial flow with a triangular microstructure, which creates a vortex regardless of the flow regime (Reynolds number), allows for a wide range of flow rates. Through self-assembly and precipitation, the chip creates a variety of nanoparticles for gene and protein delivery, including polyplexes, lipid nanoparticles, and solid polymer nanoparticles. It has also been successful in expressing GFP plasmid DNA in human T cells [140].

### 13.3. Three-Dimensional Printing Approaches to Develop Complex Composite Biomaterials for Local Delivery

Scaffolds made of polysaccharides have been successfully used to replicate the circumstances that encourage the regeneration of skin tissue after damage. The development of scaffolds with controlled and repeatable macro- and micro-structures that improve the quality of restored tissue and encourage spontaneous repair is made easier with three-dimensional (3D) advanced additive manufacturing technology. However, as seen in diseases like diabetes, vascular problems, chronic infections, and others, the body’s capacity to mend tissue is reduced when chronic inflammation occurs. In these circumstances, using growth factors and other therapeutic adjuncts in addition to standard treatments is a viable way to speed up the healing of skin lesions. Carefully designed polysaccharide scaffolds made via 3D printing provide a robust structure that can be applied to the controlled release of bioactive substances. Biopolymers known as human elastin-like polypeptides (HELPs) react to stimuli. They are attractive compounds for creating composites with intelligent properties because of their architecture, making it easier to incorporate domains with biological functions. This study combined the HELP components with 3D-printed scaffolding made of chitosan and alginate. The bioactive component, epidermal growth factor (EGF), was conjugated with the HELP biopolymer. These clever bioactive composites are suitable for developing multifunctional dressings that enable the localized release of medicinal substances, according to the development of various structures and the evaluation of their stimuli-responsive behavior and biological activity [141].

### 13.4. Current Challenges of Nano-Drug Delivery Strategies

Bone metastasis presents a particularly challenging environment for nano-drug delivery systems due to the unique physiology of bone tissue and the complexity of the metastatic microenvironment. One major challenge is crossing the blood–bone-marrow barrier, combined with the inherently low vascular perfusion of bone compared to other tissues. Systemically administered nanoparticles must navigate the sinusoidal capillary fissures, which are approximately 80–100 nm in diameter, and the dense mineralized matrix to reach the metastatic sites12. The bone microenvironment demands precise targeting to avoid off-target effects and achieve sufficient local drug concentration. Strategies employing bone-targeting moieties like bisphosphonates or small peptides that bind hydroxyapatite have been developed; however, their strong binding affinity can sometimes hinder drug release, leading to a trade-off between targeting efficiency and controlled release [142].

Another significant challenge lies in the engineering and design of the nanocarriers themselves. The formulation of nanoparticles that can encapsulate, protect, and then deliver a therapeutically relevant concentration of drugs is hindered by issues such as stability, rapid clearance by the reticuloendothelial system, and potential immunogenicity [143]. These particles are often modified with targeting ligands (e.g., RGD peptides) to mimic natural homing mechanisms and enhance bone-seeking properties; however, optimizing the balance between effective targeting, drug loading capacity, and release kinetics in such a complex environment remains challenging [144]. Many nano-delivery systems face difficulties in overcoming multidrug resistance and in reliably delivering combination therapies aimed both at inhibiting cancer cell proliferation and controlling the associated osteolytic activity [145]. These challenges are compounded by the heterogeneous nature of the tumor microenvironment in bone metastases, where the interplay between tumor cells and bone stromal cells complicates the uniform distribution of therapeutic agents.

## 14. Sustainability and Cost-Effectiveness

### 14.1. Environmental Impact of Nanomaterials

According to a study, despite their benefits in some applications, nanoparticles pose serious environmental and human health hazards. To clarify the harmful impacts of nanoparticles, the studies used various biological models and biomarkers, including soil samples, soybean seeds, and human bronchial epithelial cells (Beas-2). The most thoroughly studied nanoparticles are silicon dioxide, ferric oxide, cerium oxide, titanium dioxide, zinc oxide, multi-walled carbon nanotubes (MWCNTs), and single-walled carbon nanotubes (SWCNTs). According to this study, reduced cell viability, cell death, reactive oxygen species production, dose-dependent oxidative stress, DNA damage, apoptosis, and the induction of inflammatory responses are the main health impacts of nanoparticles. This research has found a significant gap in the scientific literature regarding the effects of nanoparticles on the environment across all categories. Future studies should analyze how various types of nanoparticles affect soil, marine, and terrestrial ecosystems. The results of this evaluation show that there is little data regarding the relative effectiveness of primary cells and immortalized cell lines as biomarkers in nanosafety studies. Future research should evaluate their effectiveness to determine which of these two cell types can be a reliable biomarker for nanosafety studies. Since this study shows that these nanoparticles are among the least investigated, future research in this area must examine the toxicological consequences of platinum, gold, magnesium oxide, molybdenum trioxide, tungsten trioxide, and carbon black. The results of this review will help stakeholders and policymakers assess the potential effects of nanoparticles [146].

### 14.2. Approaches to Make Nano-Delivery Systems More Affordable and Readily Available

The ultimate innovation in medicine, nanomedicine, is a prime example of the powerful fusion of medical research and nanotechnology. This multidisciplinary field uses nanotechnology to manipulate and control materials at the nanoscale, which improves the development of highly targeted and effective drug delivery systems. The growing need for safe and effective treatments for a variety of illnesses, including cancer, heart disease, and neurological diseases, is reflected in the growing market for nanomedicine. The versatility of nanomedicine is demonstrated not only in drug delivery, but also in tissue engineering, biomaterials, and diagnostic imaging. 

Drug delivery systems, tissue engineering, and individualized therapy are all expected to be greatly impacted by the development of nanomedicine technology. These advancements will improve organ transplantation and repair results, provide more effective, individualized treatment plans for patients, and enhance drug absorption while reducing dosages and toxicity [147,148,149].

However, nanomedicine has many hazards and challenges, including regulatory monitoring, biosafety, and industrial uniformity. It is difficult to guarantee consistent quality in large-scale manufacture, which could impact the effectiveness and caliber of medications. A lack of a unified regulatory framework and the need to ensure the safety of nanomedicine products could lead to insufficient or overly stringent regulations, which would hinder development and market accessibility. Standardized production processes, stringent biosafety evaluations, and extensive regulatory frameworks are necessary to address these problems. Enforcing strict production procedures, supervision guidelines, and quality assurance measures will ensure the consistency and superiority of the final product. Furthermore, continuous risk monitoring and proactive problem solving are necessary to enhance biological safety assessments of nanomedicine products. A thorough regulatory framework must be established to ensure compliance and preserve scientific integrity in market access, and international collaboration must be strengthened. These strategies will significantly impact the development and application of nanomedicine [149].

The fields of illness prevention, diagnosis, and therapy could all be revolutionized by nanomedicine. Nanotechnology improves patient outcomes, decreases toxicity, and increases therapeutic precision. Notably, NPs can target tumors specifically and penetrate biological barriers, allowing for more effective cancer treatments while protecting healthy tissue. Ongoing research and development highlight its potential to treat significant medical issues and enhance world health. A SWOT analysis reveals the high clinical potential of nanomedicine. One particularly exciting field is regenerative therapy, where organ engineering is greatly advanced by nanotechnology, notably in organ transplantation [150,151].

When it comes to clinical applications of nanomedicine, a SWOT analysis provides a strategic framework that helps researchers to address challenges and threats while using strengths and opportunities. Researchers, doctors, and regulatory agencies must collaborate and use interdisciplinary approaches to advance nanomedicine. We can accelerate the development of safe and efficient nanomedicines by combining these diverse fields of expertise, enabling cutting-edge medical treatments that significantly enhance human health and well-being [152].

## 15. Conclusions

Bone-targeted nanomedicine offers a paradigm shift for managing skeletal metastases by concentrating therapeutic payloads within the tumor–bone interface while sparing healthy organs. Emerging carriers exploit physicochemical tuning, bone-affine ligands, and stimuli-responsive release to outperform conventional bisphosphonates, radiotherapy, and systemic chemotherapy. However, translation remains hampered by heterogeneous EPR effects, incomplete toxicology, and scant clinical evidence. Robust in vivo imaging, immunocompetent models, and early-phase trials are critical next steps. Integrating enzyme-shielding strategies for difficult molecules such as anandamide, and coupling nanocarriers with immuno- or radiotherapies, may unlock synergistic control of tumor growth and skeletal morbidity. Continued multidisciplinary collaboration will be crucial to convert nano-DDS promise into routine care. Although this review offers a comprehensive catalogue of nano-drug delivery systems for bone metastasis, it underplays their clinical translation, for example pivotal details on ongoing Phase I–III trials, regulatory chemistry manufacture control requirements, and cost–utility projections are largely absent.

## Figures and Tables

**Table 1:** Comparative landscape of nano-based drug delivery platforms targeting bone metastasis.

Platform	Typical Composition/Architecture	Key Advantages for Bone Targeting	Main Limitations	Representative Example
Liposomes	Phospholipid bilayer vesicles; often PEG-, chitosan-, glucose-, or Au-nanorod-coated	Biocompatible, load hydro- and lipophilic drugs; stimuli-responsive; surface easily modified	Instability, drug leakage, scale-up issues	Glu-decorated paclitaxel liposomes outperform free PTX in bone lesions
Polymeric NPs	PLGA, PLA, PCL ± chitosan, PEG, bioactive glass	Tunable size and release; FDA-accepted PLGA; scaffold embedding	Poor loading of hydrophilic drugs; harsh coupling chemistries	PLGA–chitosan NPs in bioactive glass scaffold sustain local chemo
Polymeric micelles	Self-assembled PEG-b-PCL/TPGS blocks	Solubilize hydrophobes; serum-stable; dual-drug, stimuli-responsive	Possible in vivo disassembly; limited large cargo	DOX/PTX mPEG-PαLA micelles synergistically suppress osteosarcoma
Dendrimers	Branched PAMAM cores with multivalent surface	High loading (encap. or covalent); multivalent targeting; size tunable	Complex synthesis; cationic toxicity; burst release	Folate-PAMAM-MTX conjugate shows lysosomal-triggered release
MSNs	Ordered SiO_2_ with 2–10 nm pores; gatekeeper surface chem	Very high loading; pH/redox/enzyme gating; surface tunable	RES uptake; need strict pore and gate control	β-CD-capped redox-responsive DOX-MSNs delay bone lesions
Metal/metal-oxide NPs	Au spheres/rods, Pt, TiO_2_, etc.	Photothermal, imaging-therapy duality; easy conjugation	Poor biodegradability; off-target heating	PDA-coated Au NPs deliver DOX and trigger immunogenic death
SPIONs	Fe_3_O_4_ core with polymer/dendrimer shell	MRI contrast, magnetic targeting, hyperthermia	Heterogeneous tumor penetration; receptor variability	FA-PAMAM SPIONs ferry curcumin for MRI-guided therapy
Hybrid systems	Organic–inorganic composites (e.g., PDA-ZrO_2_, HMONs)	Combine stability + biodegradability; multimodal imaging; tumor-specific degradation	Synthetic complexity; limited tox/clearance data	Disulfide-linked HMONs degrade reductively for bone chemo-photo

**Table 2:** Stimuli-responsive nano-DDS tailored for bone metastasis.

Stimulus/Trigger	Representative Nanocarrier (Design Feature)	Therapeutic Payload	Release Mechanism and Bone-Specific Advantage	Key Pre-Clinical Outcome
pH + GSH (redox) dual	Ammonium-salt-gated mesoporous silica nanoparticle (MSN) with amide + disulfide linkers	Doxorubicin/siRNA	Acidic tumor pH hydrolyzes the amide bond; high intracellular GSH cleaves -S-S- to open pores	There is >80% drug release in bone-mimicking acidic/reducing milieu; minimal leakage in serum
pH + GSH dual + bone ligand	Alendronate-decorated core loaded with chemo-agent	Paclitaxel	Bone-affine alendronate guides NP to lesions; acidic and GSH gradients unlock carrier	Three-fold higher drug in tibial lesions vs. free PTX; marked osteolysis reduction
Mild hyperthermia (NIR)	Thermo-sensitive liposome + gold nanorod hybrid (TSL-GNR)	Paclitaxel	NIR raises local temp ≈ 42–45 °C → lipid phase transition → on-demand release	Eliminated metastatic foci, restored osteoclast/osteoblast balance, extended survival in mice
Alternating magnetic field	SPION-based vesicle with lipid shell (SPFeNOC concept)	Curcumin/reactive-oxygen pro-drug	External field heats membrane, increases permeability; magnet guides NP to marrow	Dual MRI/therapy; precise intramedullary release with chemodynamic tumor kill
Focused ultrasound (FUS)	ThermoDox^®^-like DOX liposomes applied to rabbit femur	Doxorubicin	FUS raises bone marrow temp ≈ 41 °C → rapid payload burst	An 8- to 17-fold DOX enrichment in marrow vs. non-heated control
